# Into the second decade

**DOI:** 10.1098/rsob.210378

**Published:** 2022-01-12

**Authors:** Jon Pines

**Affiliations:** Cancer Biology, Institute of Cancer Research, London SW3 6JB, UK

## Abstract

*Open Biology* is 10 years old and we have much to celebrate. *Open Biology* launched as the Royal Society's first fully online, open access journal dedicated to cell and molecular biology. The underlying principle of *Open Biology* is to enable discoveries to be quickly and easily disseminated through the community, and in this vein in the first 10 years of the journal we have introduced format-free submission, mandated open peer review where the reviews and author responses are published with the paper, and established our enthusiastic Preprint Team under the guidance of Prof. Michael Ginger. Credit for most of this success is due to the guiding hand of David Glover, our founding editor, and the team at Royal Society Publishing.

Our first decade established *Open Biology* as a highly visible place to publish original research. Our most cited article on PINK1 phosphorylation of Parkin from Miratul Muqit in 2012 has been cited 679 times, and the journal Impact Factor is now 6.41 (https://royalsocietypublishing.org/rsob/citation-metrics). *Open Biology* has also built a reputation for authoritative reviews. Of particular note are our commemorative articles on Antony van Leeuwenhoek and on Centrosome Biology, and our special feature on Protein Fatty Acylation. Our next special feature will be on ‘Advances in Quantitative Bioimaging' and will be edited by Ricardo Henriques, Aubrey Weigel, Christoph Leterrier and Ilaria Testa. I for one am greatly looking forward to catching up with this exciting and rapidly moving area of science.

We intend to build upon our success to continue our quest to make publishing your research less frustrating and more constructive, but we also aim to bring back the excitement of the free exchange of ideas. To this end, we introduced our *Opening Up* section to research papers as a place to speculate and set out your ideas in broader context, and we are delighted to see that other journals are following our lead. Our *Open Questions* articles focus on important unanswered questions or identify neglected but important areas of study, and we have just introduced a *Short Communication* article format to provide a format to draw attention to new and challenging ideas. We will soon introduce tools for readers to comment upon articles on-line, and we are planning a series of moderated on-line discussions to debate major topics and issues in contemporary research. I encourage you to let us know of any subjects that you would like to see critically examined and debated.

Thus, as *Open Biology* begins its second decade, as befits a journal guided by active scientists, we are well on the way to making it the natural home for exciting, innovative science, for challenging ideas, and for robust but informed debate.

